# Focal Adhesion Kinase Regulates Fibroblast Migration via Integrin beta-1 and Plays a Central Role in Fibrosis

**DOI:** 10.1038/srep19276

**Published:** 2016-01-14

**Authors:** Xue-Ke Zhao, Yiju Cheng, Ming Liang Cheng, Lei Yu, Mao Mu, Hong Li, Yang Liu, Baofang Zhang, Yumei Yao, Hui Guo, Rong Wang, Quan Zhang

**Affiliations:** 1 Department of Infectious Diseases, The Hospital Affiliated to Guizhou Medical University, Guiyang, Guizhou, China; 2 Department of Infectious Diseases, the First Hospital Affiliated to Soochow University, Suzhou, Jiangsu, China; 3 Prenatal Diagnostic Center, The Hospital Affiliated to Guizhou Medical University, Guiyang, Guizhou, China

## Abstract

Lung fibrosis is a major medical problem for the aging population worldwide. Fibroblast migration plays an important role in fibrosis. Focal Adhesion Kinase (FAK) senses the extracellular stimuli and initiates signaling cascades that promote cell migration. This study first examined the dose and time responses of FAK activation in human lung fibroblasts treated with platelet derived growth factor BB (PDGF-BB). The data indicate that FAK is directly recruited by integrin β1 and the subsequent FAK activation is required for fibroblast migration on fibronectin. In addition, the study has identified that α5β1 and α4β1 are the major integrins for FAK-mediated fibroblast migration on fibronect. In contrast, integrins αvβ3, αvβ6, and αvβ8 play a minor but distinct role in fibroblast migration on fibronectin. FAK inhibitor significantly reduces PDGF-BB stimulated fibroblast migration. Importantly, FAK inhibitor protects bleomycin-induced lung fibrosis in mice. FAK inhibitor blocks FAK activation and significantly reduces signaling cascade of fibroblast migration in bleomycin-challenged mice. Furthermore, FAK inhibitor decreases lung fibrotic score, collagen accumulation, fibronectin production, and myofibroblast differentiation in in bleomycin-challenged mice. These data demonstrate that FAK mediates fibroblast migration mainly via integrin β1. Furthermore, the findings suggest that targeting FAK signaling is an effective therapeutic strategy against fibrosis.

Organ fibrosis is a devastating disease in lung, liver, cardiovascular system, characterizing by excessive production and accumulation of extracellular matrix (ECM) proteins^1^,^2^,^3^. Fibrotic environment also contributes to cancer progression^4^,^5^. Lung fibrosis is a major medical problem for the aging population worldwide. The overall population affected by lung fibrosis is steadily increasing. Mesenchymal cells, mainly fibroblasts, are identified in normal wound healing locations and in fibrotic lesions. Fibroblast migration into wounded areas has been described as one of the initial steps affecting the consequence of wound healing and tissue remodeling. Persistent fibroblast migration contributes to the expansion of fibrotic lesions in multiple organs and results in excessive tissue remodeling, usually ends with fibrotic scar. Small aggregates of fibroblasts found in fibrotic lungs of pulmonary fibrosis patients are known as the “leading edge” of the unstoppable fibrotic responses in these patients^6^. The formation of these “leading edges” in fibrotic tissues likely results from persistent fibroblast migration stimulated by pro-fibrotic factors, such as platelet-derived growth factor BB (PDGF-BB). PDGF-BB has been implicated in many fibrotic diseases, including lung fibrosis^7^, liver fibrosis^2^, atherosclerosis^3^, and skin fibrosis^8^. PDGF-BB is well known for its function in promoting cell migration, particularly in cancer cell migration and fibroblast cell migration^9^,^10^.

Previous data demonstrate that fibroblasts derived from pulmonary fibrosis patients have increased cell migration across basement membranes when compared to normal human lung fibroblasts^11^. Increased fibroblast migration likely contributes to development of the leading edges of progressive fibrotic lesions, as well as facilitates the formation of fibrotic reticulum penetrating the surrounding tissues and extends the fibrotic scar into surrounding tissues. Cell migration is a complex and coordinated biological process. Particularly, the interaction between integrin receptors located on cell surface and ECM proteins around cells plays a critical role in promoting cell migration and directing the cell migration^10^,^11^.

Cell migration is a complex and coordinated biological process. Focal adhesion kinase (FAK) plays an essential role in cell migration. FAK is required for the signaling cascade initiated by the interaction between integrins and ECM proteins, which promotes cell migration^10^,^12^. FAK plays an important role in integrin-mediated cell migration^10^,^13^,^14^. FAK-deficient fibroblasts show significantly decreased cell migration^15^, and re-expression of FAK in FAK-deficient cells restores the cell migration^16^,^17^. FAK can be activated by integrin engagement with extracellular matrix proteins, and this results in the phosphorylation of tyrosine 397 (Y397) of FAK^10^,^13^,^16^. The phosphorylation of Y397 of FAK plays an important role in FAK-mediated cell migration^10^,^13^,^16^,^18^. Expression of the mutated Y397F FAK effectively inhibits FAK-mediated cell migration^10^,^19^,^20^. Increased FAK expression and increased Y397 phosphorylation of FAK have been found in many tumor tissues, and in tumor cell lines that show an increased cell migration rate^21^,^22^. Increased tumor cell migration is involved in tumor progression and invasion^23^. Fibronectin (FN) is excessively produced and abundantly exists in fibrotic lesions. FN likely contributes to FAK activation during fibrotic remodeling. However, little information is currently available regarding the specific integrin receptor(s) involved. As FAK senses the extracellular stimuli and initiates signaling cascades which are important for cells to correctly response to the extracellular stimuli under different situations^10^, targeting FAK signaling may interrupt the persistent fibrosis.

In this study, we show that pro-fibrotic factor, platelet derived growth factor BB (PDGF-BB), induces FAK activation and lung fibroblast migration. We provide evidences that FAK is directly associated with integrin β1 in PDGF-BB stimulated lung fibroblasts. The association of FAK and integrin β1 is important for PDGF-BB stimulated fibroblast migration on FN. Our results further demonstrate that FAK activation is a critical step in mediating fibroblast migration. The association of FAK and integrin β1 is required to activate FAK. Integrins α5β1 and α4β1 contribute to about 85% effects of FAK-mediated fibroblast migration on FN. In contrast, integrins αvβ3, αvβ6, and αvβ8 contribute to about 15% effects of FAK-mediated fibroblast migration on FN. FAK inhibitor significantly reduces PDGF-BB stimulated fibroblast migration. Importantly, our data demonstrate that FAK inhibitor protects bleomycin-induced lung fibrosis in mice. FAK inhibitor significantly reduces FAK activation and significantly inhibits signaling molecules of cell migration cascade, including Rac activation and S100A4 expression, in bleomcyin-challenged mouse lungs. In addition, FAK inhibitor decreases lung fibrotic score, collagen accumulation, FN production, and myofibroblast differentiation. These data demonstrate that FAK mediates fibroblast migration mainly via integrin β1. Furthermore, the findings suggest that targeting FAK signaling is an effective therapeutic strategy against fibrosis.

## Results

### FAK is recruited and directly associated with integrin β1 in response to PDGF-BB treatment in fibroblasts

**Figure 1:**
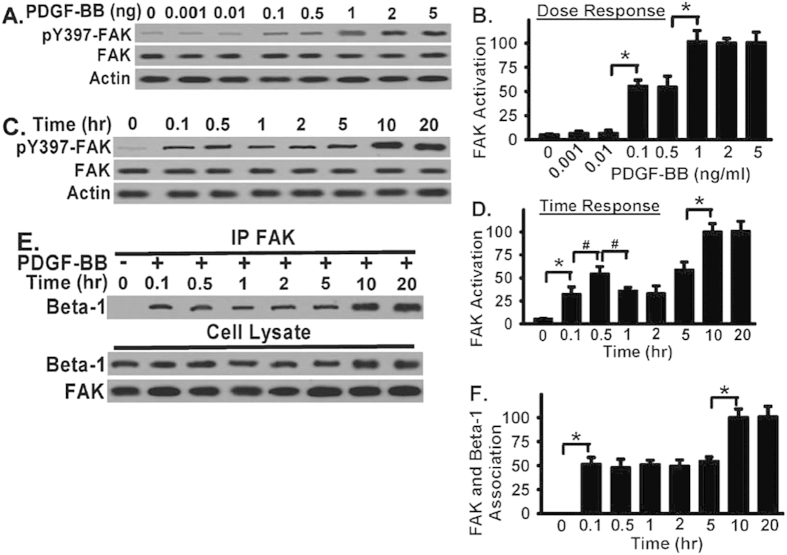
FAK is activated in a dose-dependent and time-dependent manner, and directly associated with integrin β1 in response to PDGF-BB treatment in fibroblasts. (**A**) Normal human lung fibroblasts were planted on FN (10 μg/mg), serum starved, treated with PDGF-BB at the indicated dose for overnight, and lysed. Whole cell lysates were Western blotted with indicated antibodies. FAK activation was examined by antibody toward pY397 of FAK. Beta-actin (actin) and total FAK levels were used as a loading control and served for quantification. (**B**) Densitometry of FAK activation from Panel A. pY397 of FAK levels were normalized to total FAK levels. Data were represented as the percentage of FAK activation relative to that in highest dose (5 ng/ml, set as 100%), as basal FAK activation is very low or minimal in vehicle-treated fibroblasts (bar 1). (**C**) Fibroblasts were treated as in Panel A, followed by PDGF-BB (2 ng/ml) for the indicated time points and lysed. Whole cell lysates were Western blotted with indicated antibodies. (**D**) Densitometry of FAK activation from Panel C (from a total of at least three individual tests). pY397 of FAK levels were normalized to total FAK levels. Data were represented as the percentage of FAK activation relative to that in longest time point (20 hours, set as 100%), as basal FAK activation is very low or minimal in vehicle-treated fibroblasts (bar 1). (**E**) Fibroblasts were treated as in Panel A, followed by PDGF-BB (2 ng/ml) for the indicated time points and lysed. Whole cell lysates were used for coimmunoprecipitation or pull-down with anti-FAK IgG, followed by Western blotted with anti-β1 antibody to examine the FAK-β1 interaction. Whole cell lysates were also Western blotted for total FAK and β1 integrin expression in fibroblasts. (**F**) Densitometry of FAK-β1 association from Panel E. Data were represented as the percentage of association elative to that in longest time point (20 hours, set as 100%), as the basal association is very low or minimal in vehicle-treated fibroblasts (bar 1). All above data were pooled from a total of at least three individual assays. *represents <0.01. ^#^represents <0.05.

Fibronectin (FN) is excessively produced and abundantly exists in fibrotic lesions. FN likely contributes to FAK activation during fibrotic remodeling. However, little information is currently available regarding the specific integrin receptor(s) involved. As FAK senses the extracellular stimuli and initiates signaling cascades which are important for cells to correctly response to the extracellular stimuli under different situations^10^, we first examine the interaction between FAK and FN receptor integrin β1 in response to one important pro-fibrotic factor, platelet-derived growth factor BB (PDGF-BB), in fibroblasts. Human lung fibroblasts were serum starved, plated into FN-coated (10 ug/ml) tissue culture wells, and subjected to Western blot assays for FAK activation. FAK activation was evaluated through tyrosine 397 phosphorylatio of FAK (pY397 of FAK), a well known marker for FAK activation^10^. FAK activation induced by PDGF-BB is dose-dependent and time-dependent. PDGF-BB-induced FAK activation reached two peaks, one is at the range of 0.1–0.5 ng/ml and the other peak is at the range of 1–5 ng/ml (Fig. 1A,B). In contrast, PDGF-BB did not induce significant FAK activation in fibroblasts when PDGF-BB is less than 0.01 ng/ml (Fig. 1A,B). FAK was activated as early as 6 minutes after PDGF-BB stimulation at 2 ng/ml dose in human lung fibroblasts (Fig. 1C,D). An early but small FAK activation was shown 30 minutes after PDGF-BB stimulation (Fig. 1C,D). FAK activation is further increased 5 hours after PDGF-BB treatment and reached a peak at 20 hours after PDGF-BB treatment (Fig. 1C,D).

Although it is known that FAK can be activated by interaction with integrins^10^, the specific role of FN integrin β1 on FAK activation in the context of pro-fibrotic response has not been particularly investigated. We investigated the interaction among FAK and integrin β1 by using coimmunoprecipitation or pull-down assays (Fig. 1E). Human lung fibroblasts were serum starved, treated with PDGF-BB (2 ng/ml), and whole cell lysates were immunoprecipitated or pull-down with specific anti-FAK antibody at indicated time points (Fig. 1E). The findings indicate that FAK was not associated with integrin β1 without PDGF-BB stimulation in fibroblasts (Fig. 1E,F). Upon PDGF-BB stimulation, FAK was directly associated with integrin β1 and the association was peaked 10 hours after PDGF-BB stimulation (Fig. 1E,F). These data demonstrate that FAK is activated by PDGF-BB in a dose-dependent and time-dependent manner, and FAK is recruited and directly associated with integrin β1 in response to PDGF-BB treatment in fibroblasts.

### PDGF-BB-induced fibroblast migration is dose-dependent and time-dependent on FN; Inhibition of FAK activation decreases PDGF-BB-induced fibroblast migration but does not block the FAK association with FN receptor integrin β1

**Figure 2:**
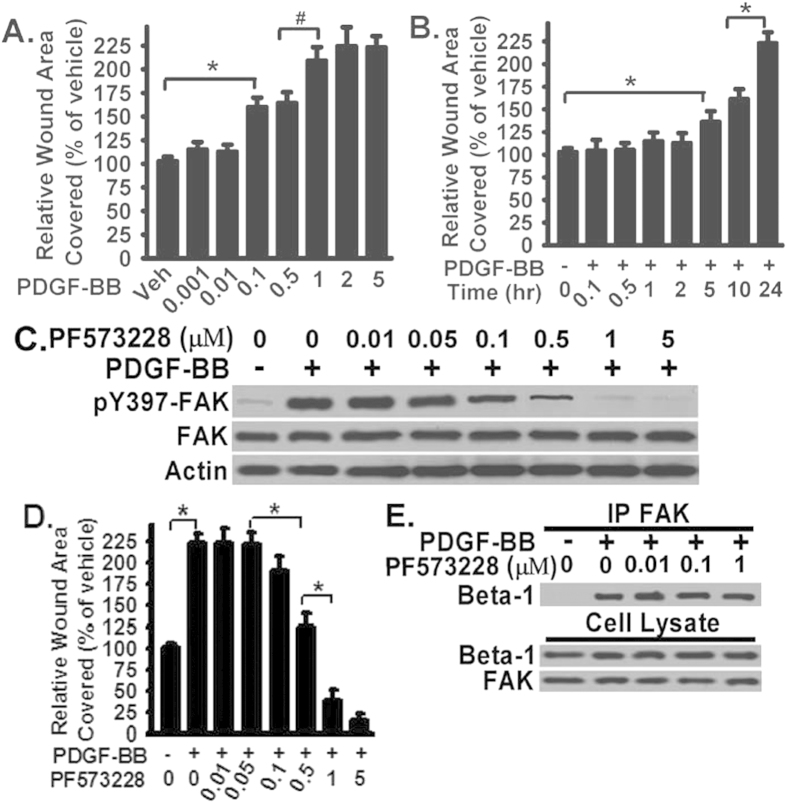
PDGF-BB-induced fibroblast migration is dose-dependent and time-dependent on FN; Inhibition of FAK activation decreases PDGF-BB-induced fibroblast migration but does not block the FAK association with FN receptor integrin β1. (**A**) Normal human lung fibroblasts were plated on FN (10 μg/mg), serum-starved, wounded, treated with PDGF-BB at the indicated dose or vehicle, and the monolayer wound area was monitored for 24 hours at 37 °C. Data were pooled from six repeats and plotted (mean + SE) as % of wound area covered over 24 hours relative to vehicle treated fibroblasts (bar 1, defined as 100%). Representative images are shown in Supplemental Fig. 1A. (**B**) Fibroblasts were treated as Panel A and with PDGF-BB (2 ng/ml) or vehicle for the indicated time points, and the monolayer wound area was monitored for 24 hours at 37 °C. Data were pooled from six repeats and plotted (mean + SE) as Panel A. Representative images are shown in Supplemental Fig. 1B. (**C**) Fibroblasts were treated as Panel A with PDGF-BB (2 ng/ml), followed by FAK inhibitor PF-573228 with indicated dose overnight, and lysed. Whole cell lysates were used for Western blot analysis with indicated antibodies. (**D**) Fibroblasts were wounded as Panel A and treated with PDGF-BB (2 ng/ml) or vehicle, followed by FAK inhibitor PF-573228 (1 μM), and the monolayer wound area was monitored for 24 hours at 37 °C. Data were pooled from six repeats and plotted (mean + SE) as Panel A. Representative images are shown in Supplemental Fig. 1C. (**E**) Fibroblasts were treated as in Panel A, followed by PDGF-BB (2 ng/ml) and FAK inhibitor PF-573228 at the indicated dose overnight, and lysed. Whole cell lysates were used for coimmunoprecipitation or pull-down with anti-FAK IgG, followed by Western blotted with anti-β1 antibody to examine the FAK-β1 interaction. Whole cell lysates were also Western blotted for total FAK and β1 integrin expression in fibroblasts. All above data were pooled from a total of at least three to six individual assays. *represents <0.01. ^#^represents <0.05.

FAK is well known for its role in promoting cell migration^10^. The above data show that FAK is recruited and directly associated with integrin β1 in response to PDGF-BB treatment in fibroblasts plated on FN (Fig. 1E,F). We next investigated the specific role of FAK association with integrin β1 on PDGF-BB stimulated fibroblast migration. First, the monolayer wound healing closure assay was used to examine the dose- and time-response of fibroblast migration on FN in response to PDGG-BB (Fig. 2). Monolayers of serum starved human lung fibroblasts were wounded, and fibroblast migration into the wounded area was documented under basal (serum free media, vehicle) and PDGF-BB-stimulated conditions. Fibroblast movement into the wounded area was tracked by real-time images, and the relative wound area covered within 24 hours analyzed (Fig. 2). PDGF-BB induced fibroblast migration in a dose-dependent manner (Fig. 2A). PDGF-BB-stimulated migration rate (wound covered area per 24 hour) is positively associated the dose used, with highest migration rate at the range of 1–5 ng/ml (Fig. 2A). PDGF-BB induced fibroblast migration is also in a time-dependent manner. The migration rate was significantly increased 5 hours and 24 hours respectively after PDGF-stimulation (Fig. 2B). The maximal fibroblast migration rate (Fig. 2A,B) is corresponding to maximal FAK activation (Fig. 1C,D) in fibroblasts.

To further understand the role of FAK-integrin β1 interaction in PDGF-BB-induced cell migration, fibroblasts were treated with or without FAK inhibitor PF-573228 (1 μM) followed by monolayer wound healing closure assay. PF-573228 inhibits PDGF-BB-induced FAK activation (pY397 of FAK) and PDGF-BB-induced fibroblast migration in a dose-dependent manner (Fig. 2C,D, respectively). PF-573228 inhibition of fibroblast migration (Fig. 2D) is closely associated with its ability to inhibit FAK activation (Fig. 2C), supporting that FAK activation is required for PDGF-BB-induced fibroblast migration. Interestingly, FAK inhibitor treatment did not block the association between FAK and integrin β1, evidenced by that PF-573228 had no effect on FAK- integrin β1 interaction (Fig. 2E), even at the dose (1 or 5 μM) which effectively inhibited PDGF-BB-induced FAK activation and fibroblast migration (Fig. 2D,E). The above data demonstrate that FAK activation in response to PDGF-BB is both dose- and time-dependent, and FAK activation is absolutely required for PDGF-BB-induced fibroblast migration. The data also suggest that FAK-integrin β1 interaction may be upstream of FAK activation, as FAK inhibitor blocks FAK activation and fibroblast migration on FN but has no effect on FAK-integrin β1 interaction.

### Integrins α5β1 and α4β1 are main integrin receptors contributing to FAK-mediated fibroblast migration and FAK activation on FN

**Figure 3:**
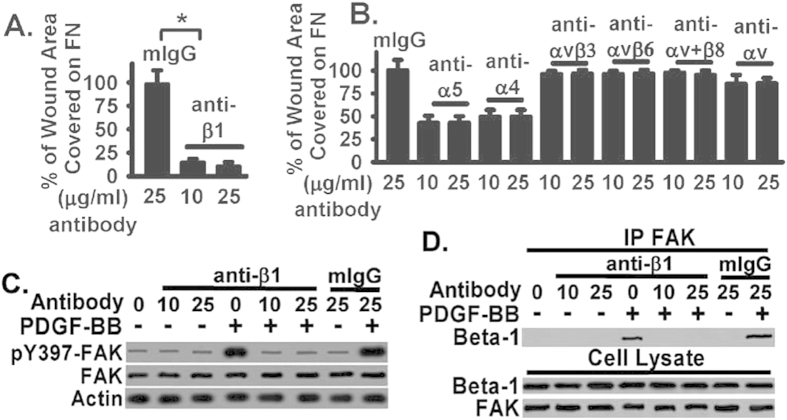
Integrins α5β1 and α4β1 are main integrin receptors contributing to FAK-mediated fibroblast migration and FAK activation on FN. (**A**) Normal human lung fibroblasts were planted on FN (10 μg/mg), serum starved, wounded as in Panel A, treated with PDGF-BB (2 ng/ml), followed by β1 integrin blocking antibody at indicated dose or control mouse IgG at the indicated dose. The monolayer wound area was monitored for 24 hours at 37 °C. Data were pooled from six repeats and plotted (mean + SE) as % of wound area covered over 24 hours relative to control IgG treated fibroblasts (bar 1, defined as 100%). Representative images are shown in Supplemental Fig. 2A. (**B**) Fibroblasts were treated as in Panel A and with PDGF-BB (2 ng/ml), followed by indicated integrin blocking antibodies or control mouse IgG at the indicated dose. The monolayer wound area was monitored for 24 hours at 37 °C. Data were pooled from six repeats and plotted (mean + SE) as % of wound area covered over 24 hours relative to control IgG treated fibroblasts (bar 1, defined as 100%). Representative images are shown in Supplemental Fig. 2B. (**C**) Fibroblasts were treated as in Panel A and lysed. Whole cell lysates were used for Western blot analysis for FAK activation. Total FAK and beta-actin (actin) levels were used for loading controls. (**D**) Fibroblasts were treated as in Panel A and lysed. Whole cell lysates were used for coimmunoprecipitation or pull-down with anti-FAK IgG, followed by Western blotted with anti-β1 antibody to examine the FAK-β1 interaction. Whole cell lysates were also Western blotted for total FAK and β1 integrin expression in fibroblasts. All above data were pooled from a total of at least three to six individual assays. *represents <0.01.

The above data (Fig. 2) suggest that FAK-integrin β1 interaction may be upstream of FAK activation, as FAK inhibitor blocks FAK activation and fibroblast migration on FN but has no effect on FAK-integrin β1 interaction. FAK contains a focal-adhesion target domain that is responsible for its docking and association with certain integrin receptors^10^. To examine whether integrin β1 interacts with FN and whether this is upstream of FAK activation, we first used blocking antibody against integrin β1 and investigated its effect on fibroblast migration on FN. Fibroblasts were plated into FN-coated tissue culture wells, serum starved, treated with integrin β1 blocking antibody or control IgG at indicated dose, and migration stimulated by PDGF-BB (2 ng/ml) was determined by monolayer wound healing closure assay. FN has multiple integrin receptors, including α5β1, α4β1, αvβ3, αvβ6, and αvβ8. Integrin β1 blocking antibody significantly blocked PDGF-BB-stimulated migration on FN (Fig. 3A). Integrin β1 blocking antibody reduced about 85% migration when compared to control IgG, p < 0.001), demonstrating that Integrin β1 is the main integrin FN receptor mediating the PDGF-BB-stimulated fibroblast migration on FN. Integrin α5 or α4 blocking antibody inhibited about 50% of fibroblast migration on FN (Fig. 3B), suggesting that α5β1 and α4β1 have overlapping functions in mediating PDGF-BB-stimulated migration. Blocking integrin αvβ3, αvβ6, or αvβ8 individually inhibited about 5% of fibroblast migration on FN (Fig. 3B), indicating that they play a minor role. This is further supported by that blocking integrin αv inhibited about 15% of fibroblast migration on FN (Fig. 3B), supporting that integrin αvβ3, αvβ6, and αvβ8 play a minor but distinct role in mediating fibroblast migration on FN.

As Integrin β1 is the main integrin FN receptor mediating the PDGF-BB-stimulated fibroblast migration on FN (Fig. 3A), we next studied the role of integrin β1 binding to FN on FAK activation. Integrin β1 blocking antibody significantly blocked PDGF-BB-stimulated FAK activation (Fig. 3C). Integrin β1 blocking antibody or control IgG had no effect on basal FAK activation in fibroblasts plated on FN (Fig. 3C, without PDGF-BB). Importantly, integrin β1 blocking antibody interrupted the association of FAK with integrin β1 in PDGF-BB-stimulated fibroblasts (Fig. 3D), demonstrating that integrin β1 binding to FN is necessary for FAK association with integrin β1 and subsequent FAK activation.

### FAK inhibitor attenuates lung fibrosis in bleomycin-challenged mice

**Figure 4:**
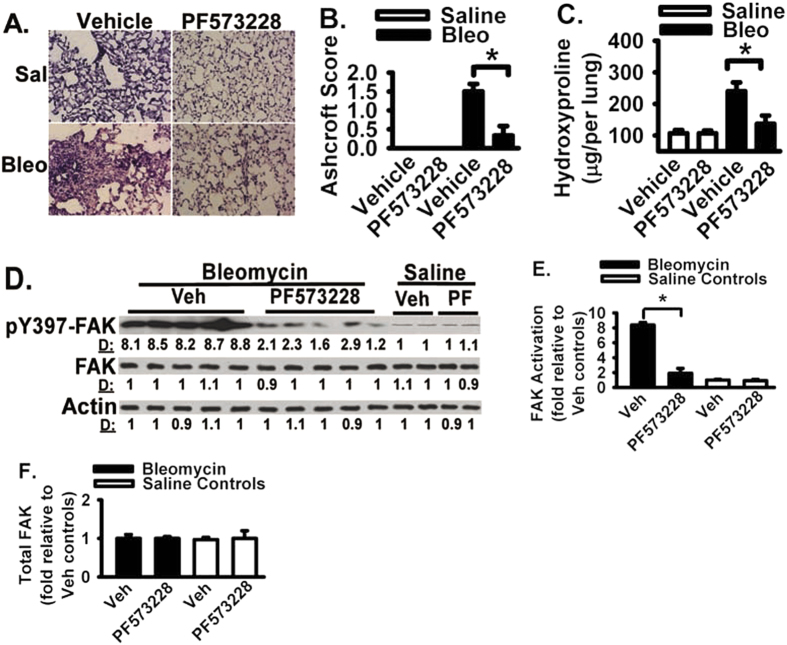
FAK inhibitor protects lung fibrosis in bleomycin-challenged mice. (**A**) Mice were intratracheally instilled with saline (Sal) or bleomycin (Bleo) (3 U/kg) and followed by daily treatment of FAK inhibitor PF-573228 (50 mg/kg, i.p. injection) or vehicle. Lungs were harvested at day 21 after bleomycin instillation, sectioned, and HE stained (200X). Representative images were shown. (**B**) The severity of lung fibrosis was examined morphometrically and represented by Ashcroft Score. (**C**) Lung hydroxyproline level was measured from vehicle-challenged and bleomycin-instilled (Bleo) mice, and represented as % hydroxyproline normalized to that in vehicle-challenged mice. (**D**) Lungs were detergent lysed, and equivalent amounts of lysate were Western blotted with indicated antibodies for FAK activation, total FAK and beta-actin loading controls. (**E**) Densitometry of FAK activation from Panel D. pY397 of FAK levels were normalized to total FAK levels. Data were represented as the fold of FAK activation relative to that in the control vehicle and saline treated group. (**F**) Densitometry of total FAK protein from Panel D. FAK levels were normalized to Actin levels. Data were represented as the fold of FAK relative to that in the control vehicle and saline treated group. Per experimental group had 8–12 mice. Data were represented as mean + SE. *represents p < 0.01.

To determine the functional role of FAK in the development of lung fibrosis, the effect of FAK inhibitor on lung fibrosis was determined by using the animal model of lung fibrosis induced by bleomycin. Bleomycin is widely used as a chemotherapy reagent but has a toxic side effect and induces lung fibrosis in human and mouse^24^. Animals were challenged with bleomcyin or saline, followed by treatment of PF-573228 or vehicle (saline) daily for 21 days. FAK inhibitor PF-573228 protects lung fibrosis in bleomycin-challenged animals (Fig. 4A). Morphometric analysis of lung tissue sections reveals an approximately 1.2-fold decrease (p < 0.01) in fibrotic score in the bleomycin-challenged mice treated with FAK inhibitor, when compared to that in bleomycin-challenged mice treated with vehicle only (Fig. 4B, Ashcroft score). There was no difference in the minimal fibrotic score observed in the lung parenchyma between animals treated with FAK inhibitor or control vehicle in the unchallenged state (Fig. 4B). Using the quantifiable measure of hydroxyproline as a surrogate for the total collagen content in whole lung tissues, FAK inhibitor significantly decreased total collagen level in bleomycin-challenged mice when compared to that in vehicle treated mice (Fig. 4C, about a 1.1-fold decrease, or 256 μg to 123 μg decrease per lung, p < 0.01), demonstrating the FAK inhibitor significantly reduced lung fibrosis. No differences on basal collagen content were noted between animals treated with FAK inhibitor and animals treated with vehicle only (Fig. 4C) or animals in unchallenged/basal conditions (data not shown). FAK activation is significantly increased in response to bleomycin challenge when compared to vehicle saline treatment (Fig. 4D, lanes 1–5 versus lanes 11–12). FAK inhibitor PF-573228 significantly reduced FAK activation in bleomycin-treated lungs (Fig. 4D, lanes 6–10 versus lanes 1–5), supporting that FAK inhibitor blocks FAK activation and attenuates lung fibrosis in animal model of lung fibrosis.

### FAK inhibitor decreases activation and expression of proteins that mediate cell migration and myofibroblast differentiation *in vivo*

**Figure 5:**
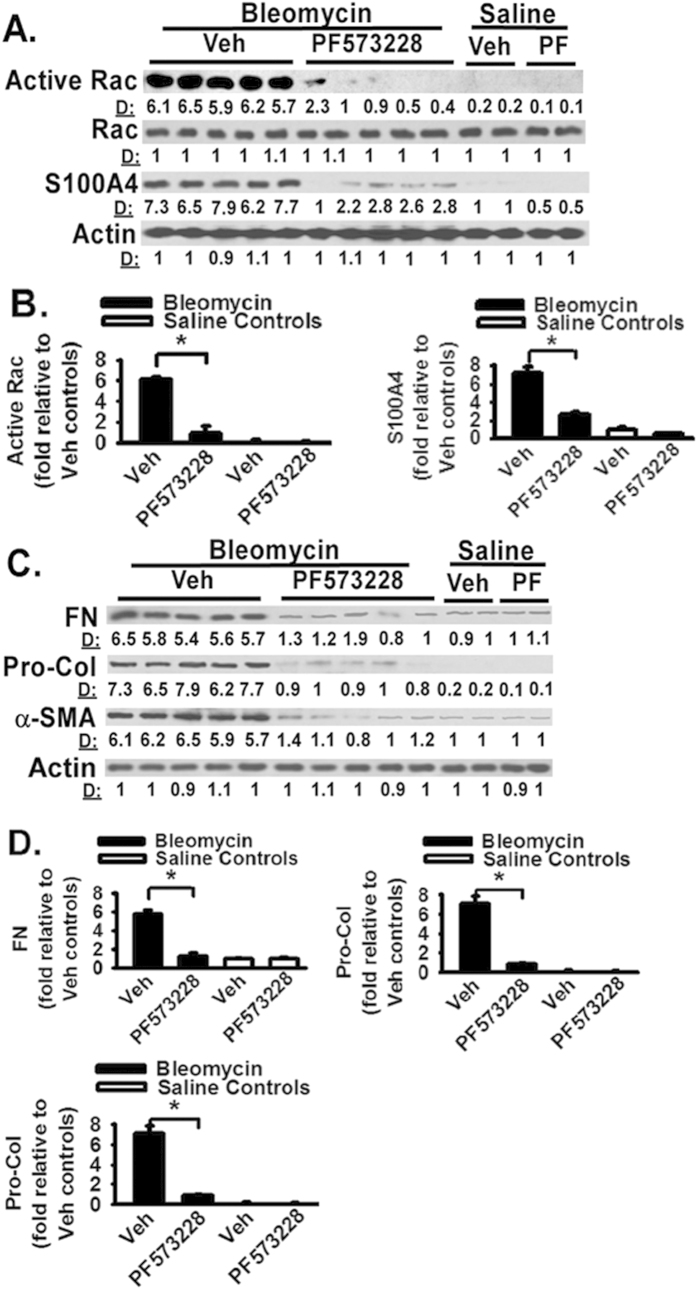
FAK inhibitor decreases activation and expression of proteins that mediate cell migration and myofibroblast differentiation *in vivo*. (**A**) Mice were intratracheally instilled with saline (Sal) or bleomycin (Bleo) (3 U/kg) and followed by daily treatment of FAK inhibitor PF-573228 (50 mg/kg, i.p. injection) or vehicle (Veh). Lungs were harvested at day 21 after bleomycin instillation, detergent lysed, and equivalent amounts of lysate were Western blotted with indicated antibodies. Per lane represents individual animals (8–12 animals per group). Representative images were shown. (**B**) Relative quantification of findings in Panel (**A**). (**C**) Mice were treated as described in Panel A. Lungs were harvested at day 21 after bleomycin instillation, detergent lysed, and equivalent amounts of lysate were Western blotted with indicated antibodies. Per lane represents individual animals (8–12 animals per group). Representative images were shown. (**D**) Relative quantification of findings in Panel (**C**).

It is likely that FAK inhibitor protects lung fibrosis by blocking multiple pro-fibrotic pathways and mechanisms in bleomycin-challenged mice. As above data support that FAK plays an essential role in fibroblast migration, one possible underlying mechanism for the protective effect of FAK inhibitor is a decrease in mesenchymal cell or fibroblast migration. We therefore assessed the effect of FAK inhibitor on the activity/expression of key cell migration regulatory proteins *in vivo* (i.e., FAK, Rac, and S100A4). S100A4 is also known as metastasin and promotes cell migration and cancer metastasis^25^. We first noted that activation of FAK (pY-397 of FAK) and Rac (GTP-bound form), and increased S100A4 expression, are part of the fibrogenic process, as their active form (FAK and Rac) or expression (S100A4) is increased in bleomycin challenged mice when compared to non-fibrotic, saline-challenged controls (Figs 4D and 5A, respectively). FAK inhibitor significantly decreased FAK activation (Fig. 4D) and active Rac (Fig. 5A) in bleomycin-challenged mice when compared to vehicle treated mice. FAK inhibitor significantly reduced S100A4 expression in bleomycin-challenged mice when compared to vehicle treated mice (Fig. 5A). Whole lung fibronectin and procollagen-1 levels after bleomycin challenge are increased in mouse lung tissues when when compared to that in saline-treated control mice (Fig. 5B, lanes 1–5 versus lanes 11–12). FAK inhibitor significantly decreased the whole lung fibronectin and procollagen-1 levels in bleomycin-challenged mice when compared to vehicle treated mice (Fig. 5B, lanes 1–5 versus lanes 6–10). In addition, FAK inhibitor significantly reduced α-smooth muscle actin (SMA) expression, a marker for myofibroblast, in bleomycin-challenged mice when compared to vehicle treated mice (Fig. 5B, lanes 1–5 versus lanes 6–10). These data demonstrate that FAK inhibitor blocks activation and expression of proteins that mediate cell migration and myofibroblast differentiation *in vivo*.

## Discussion

Our study demonstrates that PDGF-BB induces FAK activation and lung fibroblast migration in a dose-dependent and time-dependent manner. Upon PDGF-BB stimulation, FAK is quickly and directly associated with integrin β1 in PDGF-BB stimulated lung fibroblasts plated on FN. The association of FAK and β1 integrin is essential for FAK activation and FAK-mediated fibroblast migration. Our results first time show that FAK mediates PDGF-BB stimulated fibroblast migration largely via integrins β1. Integrin α5β1 and α4β1 contribute to about 85% of this effect and their function is overlapped. Integrin αv is also FN receptor but plays a minor role in FAK-mediated fibroblast migration on FN. Integrins αvβ3, αvβ6, and αvβ8 contribute to about 15% effects of FAK-mediated fibroblast migration on FN. FAK inhibitor significantly reduces PDGF-BB stimulated fibroblast migration. Importantly, our data demonstrate that FAK inhibitor protects bleomycin-induced lung fibrosis in mice. These data demonstrate that FAK mediates fibroblast migration mainly via integrin β1 and targeting FAK signaling is an effective therapeutic strategy against fibrosis.

Changes in lung fibroblast migration are thought to contribute to the pathogenesis of pulmonary fibrosis^26^,^27^. Small aggregates of fibroblasts found in fibrotic lungs of pulmonary fibrosis patients are known as the “leading edge” of the unstoppable fibrotic responses in these patients^6^, supporting that dysregulated fibroblast migration contributes to the development of fibrotic lesions and expansion of the fibrotic lesions. Persistent development and expansion of fibrotic lesions leads to persistent fibrotic remodeling in affected organs. PDGF-BB is a pro-fibrotic factor. PDGF-BB has been implicated in many fibrotic diseases, including lung fibrosis^7^, liver fibrosis^2^, atherosclerosis^3^, and skin fibrosis^8^. PDGF-BB is well known for its function in promoting cell migration, particularly in cancer cell migration and fibroblast cell migration^9^,^10^. The PDGF-BB level is increased in fibrotic response^2^,^3^,^7^,^8^; therefore, the formation of these “leading edges” in fibrotic tissues likely results from persistent fibroblast migration stimulated by PDGF-BB).

Previous data demonstrate that fibroblasts derived from pulmonary fibrosis patients have increased cell migration across basement membranes when compared to normal human lung fibroblasts^11^. Increased fibroblast migration likely contributes to development of the leading edges of progressive fibrotic lesions, as well as facilitates the formation of fibrotic reticulum penetrating the surrounding tissues and extends the fibrotic scar into surrounding tissues. Cell migration is a complex and coordinated biological process. Particularly, the interaction between integrin receptors located on cell surface and ECM proteins around cells plays a critical role in promoting cell migration and directing the cell migration^10^,^11^; however, their functions in fibrosis have not been completed revealed. TGF-β1 is known as a major fibrotic mediator by increasing ECM protein accumulation, and it regulates lung fibrosis through signaling pathways mediated by FAK and Src^28^,^29^. TGF-β1 is less potent to induce cell migration but stimulates myofibroblast phenotype and epithelial to mesenchymal transition^30^,^31^. PDGF-BB has been shown to increase cell migration in primary lung cells isolated from pulmonary fibrosis patients^32^.

FAK plays an essential role in cell migration^10^. Increased FAK activation is known to promote cell migration through regulation of focal adhesion turnover and cytoskeleton re-organization^10^,^33^. The dose- and time-response of FAK activation in the context of lung fibroblast migration on FN has not been studied previously. The data show that PDGF-BB-induced fibroblast migration is dose-dependent and time-dependent on FN. Fibroblast migration rate is positively associated the PDGF-BB dose and time (Fig. 2A,B). The findings suggest that fibroblast migration could serve to develop new fibrotic lesions or expand the fibrotic lesions once there are increased PDGF-BB in the environment.

FAK is required for the signaling cascade initiated by the interaction between integrins and ECM proteins, which promotes cell migration^10^,^12^. In fact, FAK plays an important role in integrin-mediated cell migration^10^,^13^,^34^. Our data demonstrate that FAK activation is dependent upon the PDGF-BB dose, as well as the treated time (Fig. 1A,D). The data indicate that FAK is quickly recruited and directly associated with integrin β1 in response to PDGF-BB treatment in fibroblasts plated on FN (Fig. 1E,F). FN is excessively produced and abundantly exists in fibrotic lesions. Our data support that FN likely contributes to persistent fibrotic development through FAK activation and FAK-mediated fibroblast migration.

However, little information is currently available regarding the specific integrin receptor(s) involved. The findings demonstrate that integrins α5β1 and α4β1 are main integrin receptors contributing to FAK-mediated fibroblast migration and FAK activation on FN (Fig. 3). FN has multiple integrin receptors, including α5β1, α4β1, αvβ3, αvβ6, and αvβ8. Integrin β1 contributes to about 85% fibroblast migration on FN, while integrin αv contributes to about 15% of fibroblast migration on FN (Fig. 3). Interestingly, integrin α5β1 and α4β1 both contribute to about 50% of fibroblast migration on FN (Fig. 3), suggesting that α5β1 and α4β1 have overlapping functions in mediating PDGF-BB-stimulated migration on FN. In contrast, integrin αvβ3, αvβ6, or αvβ8 play a minor role but they have distinct role in mediating fibroblast migration on FN (Fig. 3). Integrin β1 plays a major role in FAK activation induced by PDGF-BB (Fig. 3). Integrin β1 blocking antibody largely blocked PDGF-BB-stimulated FAK activation (Fig. 3), as well as interrupted the association of FAK with integrin β1 in PDGF-BB-stimulated fibroblasts (Fig. 3). These data demonstrate that integrin β1 binding to FN is necessary for FAK association with integrin β1 and subsequent FAK activation in response to PDGF-BB.

FAK contains a focal-adhesion target domain that is responsible for its docking and association with certain integrin receptors^10^. It is unknown whether FAK binding to integrins through its focal-adhesion domain can be uncoupled with FAK activation. FAK inhibitor PF-573228 inhibits PDGF-BB-induced FAK activation and fibroblast migration in a dose- and time-dependent manner (Fig. 1). Interestingly, FAK inhibitor treatment did not block the association between FAK and integrin β1, evidenced by that PF-573228 had no effect on FAK- integrin β1 interaction (Fig. 1E), even at the dose (1 or 5 μM) which effectively inhibited PDGF-BB-induced FAK activation and fibroblast migration (Fig. 1D,E). These data demonstrate that FAK activation is absolutely required for PDGF-BB-induced fibroblast migration. At the same time, the above data also demonstrate that FAK activation requires the integrin β1-bindign to FN and FAK-integrin β1 interaction is upstream of FAK activation.

As FAK senses the extracellular stimuli and initiates signaling cascades which are important for cells to correctly response to the extracellular stimuli under different situations^10^, targeting FAK signaling may interrupt the persistent fibrosis. To determine the functional role of FAK in the development of lung fibrosis, the effect of FAK inhibitor on lung fibrosis was determined by using the animal model of lung fibrosis induced by bleomycin. Bleomycin lung fibrosis model is controversial but is widely used and extensively published^24^,^35^,^36^,^37^. FAK inhibitor protects lung fibrosis in bleomycin-challenged animals (Fig. 4). FAK inhibitor significantly inhibited FAK activation in bleomycin-challenged mice. FAK inhibitor significantly reduced fibrotic score and total lung collagen content in bleomycin-challenged mice, supporting that FAK inhibitor is protective against the pro-fibrotic effects of bleomycin. These data supporting that FAK inhibitor blocks FAK activation and attenuates lung fibrosis in animal model of lung fibrosis. Our results confirm the anti-fibrotic effects published previously and extend to further understand the underling molecular mechanisms. It is likely that FAK inhibitor protects lung fibrosis by blocking multiple pro-fibrotic pathways and mechanisms in bleomycin-challenged mice. Our findings support that one underlying mechanism for the protective effect of FAK inhibitor is a decrease in mesenchymal cell or fibroblast migration signing in animals treated with FAK inhibitor. FAK inhibitor significantly reduced the activity/expression of key cell migration regulatory proteins (i.e., FAK, Rac, and S100A4) in bleomycin-challenged mice. Tyrosine 397 phosphorylation of FAK initiates signaling cascade that is a main driving force to promote cell migration^10^. Small Rho GTPases, such as Rac, regulate cell migration through modulating actin polymerization and lamellipodia formation^38^,^39^,^40^. Activation of Rac is increased during cell migration^38^,^39^,^40^, and Rac activation is used as one of the hallmarker of cell migration signaling cascade. The evidence in the literature suggests that Rac is a downstream intermediate of FAK-dependent cell migration^38^,^39^,^40^. S100A4 is also known as metastasin and promotes cell migration and cancer metastasis^25^. S100A4 expression in normal tissue is minimal, and increased S100A4 indicates an ongoing cell migration and metastasis *in vivo*^25^,^41^,^42^,^43^,^44^,^45^. In fact, fibrotic lung tissues (from bleomycin-challenged mice) show significant activation of FAK (pY-397 of FAK) and Rac (GTP-bound form), and significant increased S100A4 expression; therefore, they are part of the fibrogenic process (Figs 4 and 5). FAK inhibitor significantly decreased the activation of FAK and Rac, and significantly decreased S100A4 expression in bleomycin-challenged mice. These data support that FAK inhibitor decreases activation and expression of proteins that mediate cell migration and myofibroblast differentiation. Additional supports are that FAK inhibitor decreased the whole lung fibronectin and procollagen-1 levels, and reduced α-SMA expression (a myofibroblast differentiation marker) in bleomycin-challenged mice. Myofibroblast differentiation is one main driving force of fibrosis^46^,^47^. These data suggest that FAK inhibitor blocks the development of fibrotic lesions at least through inhibition of fibroblast migration, ECM protein production, and myofibroblast differentiation.

In order to design effective therapies for progressive fibrotic diseases in lung, liver, and other organs, it is important to understand the signaling pathways contributing to the progressive development of fibrotic lesions. The significance of fibroblast migration in the context of lung injury and fibrotic remodeling is continuously supported^11^,^48^,^49^,^50^,^51^. The intracellular signaling pathways that govern cell migration are complex and exhibit dynamic and reciprocal cross-talk and are context dependent^52^,^53^,^54^. In most tested systems, FAK and the small GTPases (Rac and Rho) promote cell migration through modulating both focal adhesion turnover, and the cytoskeleton reorganization necessary to generate the forces required for migration^10^,^54^,^55^,^56^. Thus, their activation can be conceived as both a marker and an effector of cell migration. This current study is limited to know whether manipulation of cell migration signaling downstream of FAK will change the cellular behavior and lung injury and repair in response to pro-fobrotic stimuli. In further studies, genetic deletion or pharmacologic blocking of key cell migration signaling will provide further evidence about the complicated disease process *in vivo*. We demonstrate that increased activation of FAK and Rac, as well as increased S100A4 expression in fibrotic lung tissues, and FAK inhibitor significantly inhibited these effects. As fibroblast migration is considered as one contributing factor in the development of progressive fibrotic lesions, we speculate that a dysregulated FAK signaling promotes fibroblast migration and that could be one mechanism for the progressive fibrosis in lung, liver, cardiovascular system, kidney, and other organs. Pharmacological reagents targeting the dysregulated FAK signaling or FAK interaction with ECM protein via specific integrins may have potential effective and therapeutic effects for fibrosis in these vital organs.

## Methods

### Reagents and Antibodies

Platelet-derived growth factor (PDGF-BB) was obtained from R&D Systems (Minneapolis, MN). The following purified polyclonal antibodies were purchased: anti-phospho-FAK [pY397] (Biosource, Camarillo, CA), anti-FAK (recognizes both FAK C-terminal and FRNK, Upstate Biotechnology, Lake Placid, NY). Purified mouse IgG was purchased from Molecular Probes (Carlsbad, CA). The following purified IgG antibodies were purchased from abcam (Cambridge, MA): beta-1 (β1), alpha-α-5 (α5), α4, αVβ3, αVβ5, αV, and β8. Purified IgG antibodies against pro-collagen, fibronectin (FN), and beta-actin were purchased from Santa Cruz Biotechnology (Dallas, Texas). PF573228 (FAK inhibitor) was purchased from Calbiochem (Calbiochem, Millipore, Billerica, MA). Anti-alpha (α)-smooth muscle actin (α-SMA) mouse IgG, fibronectin (FN), bleomycin, and other chemicals were purchased from Sigma-Aldrich (St. Louis, MO). Anti-S100A4 purified IgG was purchased from Dako (Carpinteria, CA).

### Cells and Cell Culture

De-identified primary adult normal human lung fibroblasts (NHL) (19LU) were purchased from the American Type Culture Collection (ATCC) (Manassas, VA). They were maintained and propagated in Dulbecco’s modified Eagle’s medium (DMEM) supplemented with 10% fetal bovine serum (FBS), 2 mM L-glutamine, 100 units/ml penicillin/streptomycin/gentamycin. Experiments were performed on early passages (passage 7–9).

### Western Blotting

Cells were lysed in 1% NP-40 lysis buffer containing the following inhibitors, PMSF, Aprotinin, Leupeptin, Sodium Vanadate, and TLCK. The protein concentration of the whole cell or whole lung lysate was determined by BCA kit (Pierce, Rockford, IL). Equivalent micrograms of whole cell detergent lysates were electrophoresed on a disulfide-reduced 12% SDS PAGE, transferred to Immobilon-P membrane (Millipore Corp., Bedford, MA), probed and stripped followed by re-probing with indicated antibodies, and developed with the enhanced chemiluminescent (ECL) system (Pharmacia Biotech, Piscataway, NJ). The expression of beta-actin protein was used as a loading control. For densitometric analysis of band intensity, a specific band on the ECL-developed film was subjected to densitometric analysis (Adobe Photoshop). The densitometric readings were pooled and averaged from three independent experiments. The background of densitometric reading on the ECL-developed film was subtracted.

### Cell Migration Assays

The 2-D wound closure monolayer/scratch motility assay and haptotactic cell migration assay toward FN were performed. Briefly, fibroblasts were harvested with buffered EDTA, resuspended in serum-free Dulbecco’s modified Eagle’s medium with 1% BSA, and plated into 24-well format tissue culture plates (1 × 10^5^ cells/well). At 24 hour, the monolayer was scratched, digital pictures were taken immediately after wounding/scratching, and again at the end of the assay. The digital pictures were used to calculate the areas of scratch without cells immediately after scratching and the remaining areas without cells at the end of the assay. The wound area covered by cell migration after scratching was equal to the difference between the two areas above, and was normalized to that in vehicle-treated or control fibroblast group. Mitomycin C was added to inhibit cell proliferation after PDGF-BB treatment. The haptotactic cell migration assay toward FN was performed in two-well Boyden-type chambers. Briefly, fibroblasts (4 × 10^4^ cells) in serum-free DMEM media with 1% BSA were plated onto 8 μm filters coated on the bottom surface with fibronectin (10 μg/ml), and allowed to migrate at 37 °C with 5% CO_2_ for 6 hours. Fibroblast cells on the upper filter surface were removed, and the cells (migrated to) on the lower filter surface were fixed, stained, and counted. Conditions were assayed in replicas of three or four, repeated two to four times, and the data analyzed and presented as the mean ± SE.

### Coimmunoprecipitation Assays

*Coimmunoprecipitation* analysis or pull-down assays was performed. Briefly, equivalent microgram of protein lysate from each sample was incubated with sepharose-coupled antibody toward FAK overnight at 4 degree, washed, subjected to 7.5% SDS–PAGE, transferred to Immobilon, and Western blotted with indicated antibodies.

### Animal Model of Lung Fibrosis and Analysis of Fibrosis *In Vivo*

Institutional and national guidelines for the care and use of animals were followed and all experimental procedures involving animals were approved by the IACUC (institutional animal care and use committee) of Guizhou Medical University (Permit Number: 1323092). Mice (C57Bl6, 8-11 weeks) were anesthetized and bleomycin (3 U/kg body weight in 50 μl saline) or saline vehicle alone (50 μl) was slowly instilled through airway to lungs by using an intratracheal catheter. For animals treated with FAK inhibitor, the animals were i.p injected everyday with 50 mg/kg PF-573228 based on information provided by vendor and published data. Control animals were i.p injected everyday with vehicle. The animals were euthanized and lung tissues were harvested at day 21 after bleomycin treatment. The lung tissues were used for histological and biochemical studies, and for protein and cell migration signaling analysis. To collect lung tissue for histological studies, the lung tissues were fixed in paraffin. To collect lung tissue for histological studies, the lung tissues were fixed and paraffin embedded. Analysis of whole lung protein extracts was performed. Whole lung homogenates were prepared in 1% NP-40 lysis buffer with the following inhibitors, 100 μM phenylmethanesulfonyl fluoride (PMSF), 10 μg/ml Aprotinin, 10 μg/ml Leupeptin, and 100 μM Sodium Vanadate, and 20 μg/ml TLCK using a polytron (Brinkmann Instruments, Westbury, NY). The resultant supernatants after centrifugation (14,000 × g for 20 min at 4 °C) were analyzed by Western blot analysis immediately or stored at –80 °C until used.

### Total Lung collagen Accumulation for Fibrosis Analysis

The whole lung collagen level was determined by whole lung hydroxyproline level. The harvested lungs were hydrolyzed in 6 M HCl at 110 °C for 24 hours, and the amount of hydroxyproline in the above lung acid-hydrolysates was performed by colorimetric assay as described previously^57^.

### Rac Activation Assays for Cell Migration Signaling

It is well known that Rac activation leads to increased cell migration. Rac activation was determined by the level of active Rac (GTP-bound form) in lung tissues *in vivo*. Lung tissues were harvested from control and experimental animal groups and whole lung lysates were prepared as described above. Equivalent micrograms of lysate reacted with p21-activated kinase-1 binding domain coupled to agarose (for Rac activation or active Rac) and immunoprecipitates subjected to 10% SDS-PAGE, transferred to Immobilon, and Western blotted with anti-Rac IgG. Total Rac in whole lung lysate was also determined by Western blot analysis and used to determine the ratio of Rac activation and also serve as a loading control.

### Statistical Analysis

Data were analyzed using the unpaired or paired *t*-test analysis (for comparisons between two groups) (Sigma Plot, SPSS Inc.), and expressed as means ± SE. Experiments were performed three to four times with duplicates. For animal studies, each experimental or control group contained 5 to 6 animals and repeated twice. A *p* value of <0.05 was considered statistically significant.

## Additional Information

**How to cite this article**: Zhao, X. *et al.* Focal Adhesion Kinase Regulates Fibroblast Migration via Integrin beta-1 and Plays a Central Role in Fibrosis. *Sci. Rep.* **6**, 19276; doi: 10.1038/srep19276 (2016).

## Supplementary Material

Supplementary Figures
