# Supplementary material for: Focal Adhesion Kinase Regulates Fibroblast Migration via Integrin beta-1 and Plays a Central Role in Fibrosis
Source: Sci Rep. 2016 Jan 14;6:19276. doi: 10.1038/srep19276 (PMC4725867; doi:10.1038/srep19276)
Supplement: Supplementary Figures [file srep19276-s1.doc]

**Focal Adhesion Kinase Regulates Fibroblast Migration via Integrin beta-1 and Plays a Central Role in Fibrosis**

Xueke Zhao, Yiju Cheng, Ming Liang Cheng, Lei Yu, Mao Mu, Hong Li, Yang Liu, Baofang Zhang, Yumei Yao, Hui Guo, Rong Wang, and Quan Zhang

**Supplementary Figure S1**


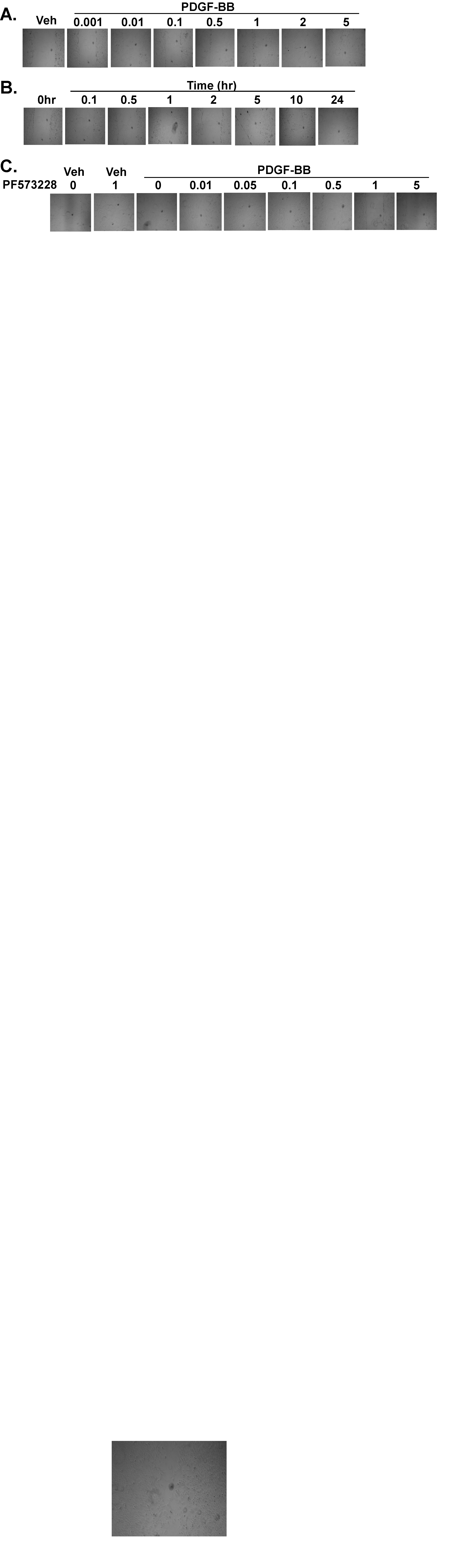


**Supplementary Figure S2**

**
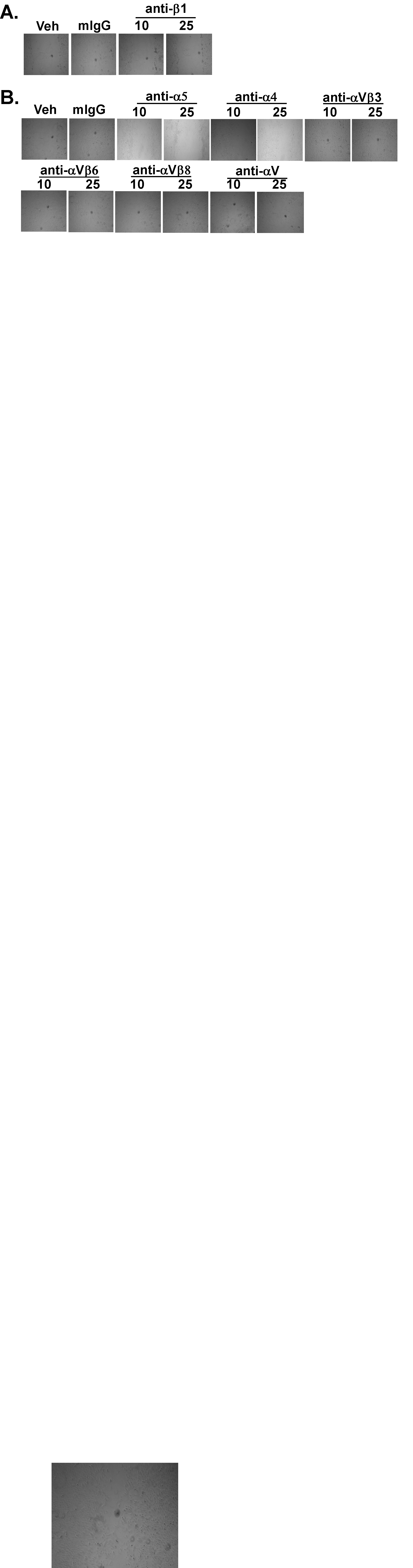
**

**Supplementary Figure Legend**

**Supplementary Figure S1**.

**PDGF-BB-induced fibroblast migration is dose-dependent and time-dependent on FN; Inhibition of FAK activation decreases PDGF-BB-induced fibroblast migration.**

**(*A):*** Normal human lung fibroblasts were plated on FN (10 µg/mg), serum-starved, wounded, treated with PDGF-BB at the indicated dose (ng/ml) or vehicle, and the monolayer wound area was monitored for 24 hours at 37°C. Representative images are shown. **(*B):*** Fibroblasts were treated as Panel A and with PDGF-BB (2 ng/ml) or vehicle for the indicated time points, and the monolayer wound area was monitored for 24 hours at 37°C. Representative images are shown. **(*C)*:** Fibroblasts were wounded as Panel A and treated with PDGF-BB (2 ng/ml) or vehicle, followed by FAK inhibitor PF-573228 (1μM), and the monolayer wound area was monitored for 24 hours at 37°C. Representative images are shown.

**Supplementary Figure S2**.

**Integrins α5β1 and α4β1 are main integrin receptors contributing to FAK-mediated fibroblast migration and FAK activation on FN.**

**(*A):*** Normal human lung fibroblasts were planted on FN (10 µg/mg), serum starved, wounded as in Panel A, treated with PDGF-BB (2 ng/ml), followed by β1 integrin blocking antibody at indicated dose or control mouse IgG at the indicated dose. The monolayer wound area was monitored for 24 hours at 37°C. Representative images are shown. ***(B):*** Fibroblasts were treated as in Panel A and with PDGF-BB (2 ng/ml), followed by indicated integrin blocking antibodies or control mouse IgG at the indicated dose. The monolayer wound area was monitored for 24 hours at 37°C. Representative images are shown.
